# Automated Assessment of the Substantia Nigra Pars Compacta in Parkinson’s Disease: A Diffusion Tensor Imaging Study

**DOI:** 10.3390/jpm11111235

**Published:** 2021-11-21

**Authors:** Niels Bergsland, Laura Pelizzari, Maria Marcella Laganá, Sonia Di Tella, Federica Rossetto, Raffaello Nemni, Mario Clerici, Francesca Baglio

**Affiliations:** 1 IRCCS, Fondazione Don Carlo Gnocchi ONLUS, 20148 Milan, Italy; nbergsland@dongnocchi.it (N.B.); mlagana@dongnocchi.it (M.M.L.); sditella@dongnocchi.it (S.D.T.); frossetto@dongnocchi.it (F.R.); rnemni@dongnocchi.it (R.N.); mclerici@dongnocchi.it (M.C.); fbaglio@dongnocchi.it (F.B.); 2 Buffalo Neuroimaging Analysis Center, Department of Neurology, Jacobs School of Medicine and Biomedical Sciences, University at Buffalo, State University of New York, Buffalo, NY 14203, USA; 3 Department of Pathophysiology and Transplantation, Università degli Studi di Milano, 20122 Milan, Italy

**Keywords:** Parkinson’s disease, MRI, substantia nigra, diffusion tensor imaging

## Abstract

The substantia nigra (SN) pars compacta (SNpc) and pars reticulata (SNpr) are differentially affected in Parkinson’s disease (PD). Separating the SNpc and SNpr is challenging with standard magnetic resonance imaging (MRI). Diffusion tensor imaging (DTI) allows for the characterization of SN microstructure in a non-invasive manner. In this study, 29 PD patients and 28 healthy controls (HCs) were imaged with 1.5T MRI for DTI. Images were nonlinearly registered to standard space and SNpc and SNpr DTI parameters were measured. ANCOVA and receiver operator characteristic (ROC) analyses were performed. Clinical associations were assessed with Spearman correlations. Multiple corrections were controlled for false discovery rate. PD patients presented with significantly increased SNpc axial diffusivity (AD) (1.207 ± 0.068 versus 1.156 ± 0.045, *p* = 0.024), with ROC analysis yielding an under the curve of 0.736. Trends with Unified Parkinson’s Disease Rating Scale (UPDRS) III scores were identified for SNpc MD (rs = 0.449), AD (rs = 0.388), and radial diffusivity (rs = 0.391) (all *p* < 0.1). A trend between baseline SNpr MD and H&Y change (rs = 0.563, *p* = 0.081) over 2.9 years of follow-up was identified (*n* = 14). In conclusion, SN microstructure shows robust, clinically meaningful associations in PD.

## 1. Introduction

The development and validation of in vivo biomarkers of Parkinson’s disease (PD), the second most common neurodegenerative disease, has continued to remain a highly active area of research [1]. Although the exact cause of PD remains unknown, it is well-established that the destruction of the dopaminergic neurons begins early on in the substantia nigra (SN) pars compacta (SNpc). In fact, at the time of diagnosis, it is estimated that between thirty to fifty percent of such neurons have already been destroyed [2]. Due to its sensitivity to different tissue properties and lack of ionizing radiation, magnetic resonance imaging (MRI) offers the possibility to investigate various aspects of pathological processes associated with the disease. For example, elevated iron deposition [3,4,5] and decreased microstructural integrity [3,6,7,8] have both been reported within the SN using different MRI modalities.

Diffusion tensor imaging (DTI) is a post-processing technique that utilizes diffusion-weighted imaging for characterizing the movement of water molecules. Although frequently used to analyze the white matter, a number of studies have highlighted its utility for studying the microstructural properties of the gray matter as well. However, reported imaging findings have not been fully consistent. On the one hand, some authors have found that DTI-derived parameters within the SN are highly affected by PD and may even serve as a potential biomarker, with one study reporting 100 percent sensitivity and specificity [9]. A recent meta-analysis supports this finding, with fractional anisotropy (FA) and mean diffusivity (MD) being significantly altered in PD patients compared to healthy controls (HC) [10]. On the other hand, a separate systematic review with corresponding meta-analysis suggested that increases in mean diffusivity (MD) are inconsistent, while fractional anisotropy (FA) is not significantly different between PD patients and HCs [11].

Although there are a number of factors that almost certainly contribute to the heterogeneity of findings in the literature, including applied sequences/acquisition parameters and patient characteristics, region of interest (ROI) placement likely plays a key role as well. For example, many studies have considered the SN as a single structure [12,13,14], despite the disease having very distinct effects on its two sub-structures. As already mentioned, the SNpc shows massive neurodegeneration already at the time of diagnosis while the SN pars reticulata (SNpr) is relatively spared until much later in the disease [15]. Thus, any imaging assessment that does not distinguish between the SNpc and SNpr is likely to inaccurately capture the underlying pathology.

With this background, we aimed to separately assess the SNpc and SNpr in a group of PD patients and a group of demographically matched HCs. We hypothesized that greater differences would be detected within diffusion measures of the SNpc compared to the SNpr. We also hypothesized that SNpc damage would be associated with greater clinical impairment.

## 2. Materials and Methods

### 2.1. Subjects

Between November 2016 and July 2018, 29 PD patients and 28 healthy controls (HC), group matched for age and sex, were enrolled into the study. After an average of 2.9 (standard deviation of 0.6) years, 14 patients returned for a clinical follow-up.

Inclusion criteria for PD patients were: (1) diagnosis of probable PD, according to the Movement Disorder Society (MDS) Clinical Diagnostic Criteria for PD [16]; (2) positive dopamine transporter (DaT) scan; (3) mild to moderate stages of the disease (Modified Hoehn and Yahr (H&Y) < 3); (4) time spent with dyskinesias assessed with MDS-UPDRS part IV lower than 2; and (5) stable drug therapy for those patients using either L-Dopa or dopamine agonists for the past three months. Exclusion criteria for all the participants included: (1) presenting with neurological diseases other than PD; (2) presenting with psychiatric disorders; (3) presenting with cardiovascular and/or metabolic diseases; and (4) being left-handed. The HC group comprised hospital personnel as well as family members or friends of PD patients.

All patients underwent UPDRS-III assessment, evaluation of H&Y stage, and the levodopa equivalent daily dose was calculated.

The study was approved by the IRCCS Fondazione Don Carlo Gnocchi Ethics Committee (approval number 3_1/7/2015 on 2015-07-01) and was performed in accordance with the principles of the Helsinki Declaration. All the participants provided their written and informed consent.

### 2.2. MRI Acquisition

All scans were acquired on the same 1.5T Magnetom Avanto MRI scanner (Siemens, Erlangen, Germany) with a 12-channel head and neck coil. The imaging system did not undergo any major hard- or software upgrades during the study. A diffusion-weighted echo-planar imaging sequence was acquired for all study participants with 64 non-collinear directions with *b*-value = 1500 s/mm^2^ and 2 volumes with *b*-value = 0 s/mm^2^ (repetition time = 7800 ms, echo time = 109 ms, matrix = 102×102×46, resolution = 2.5 mm^3^ isotropic, phase encoding = anterior–posterior). In addition, an additional image with *b*-value = 0 s/mm^2^ was acquired with the exact same parameters except for phase encoding = posterior–anterior.

### 2.3. MRI Assessment

**Figure 1:**
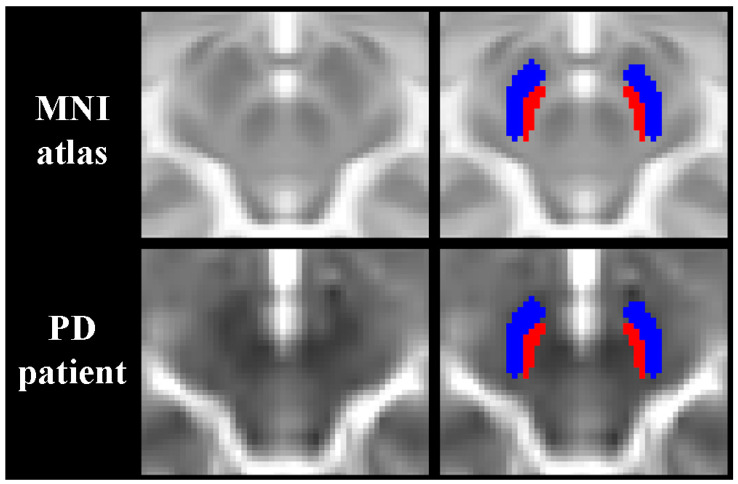
Standard space-defined regions of interest for the substantia nigra. The top row shows the standard space-defined masks of the substantia nigra, with the reticulata colored in blue and the pars compacta colored in red. The bottom row depicts a *b* = 0 image after spatial normalization to the Montreal Neuroimaging Institute (MNI) atlas in a representative Parkinson’s disease patient.

The off-resonance susceptibility-induced field was estimated from a pair of *b* = 0 s/mm^2^ images with opposite phase encoding directions using the topup tool [17]. Eddy current distortion and subject movement were then corrected using the eddy tool [18] along with automate outlier replacement [19]. The topup-derived field map was passed to the eddy tool such that a single resampling was utilized. Next, the diffusion tensor was calculated for each voxel using dtifit and scalar maps of FA, MD, axial diffusivity (AD), and radial diffusivity (RD) were calculated. The corresponding image without any diffusion weighting was subsequently warped to the T2-weighted Montreal Neuroimaging Institute (MNI) atlas using Advanced Normalization Tools (ANTs) [20], which has previously been shown to be the best performing nonlinear registration tool [21]. All registrations were visually inspected to ensure accurate spatial alignment. The DTI-derived scalar maps were then brought into MNI space using the corresponding warps. Standard space-defined probability maps of the SNpr and SNpc [22], thresholded at 0.5 and binarized, were then used to extract DTI-derived parameters (Figure 1).

### 2.4. Statistical Analysis

Statistical analyses were performed using SPSS (version 25; IBM Corp., Armonk, NY, USA). Differences in demographic characteristics between PD patients and HCs were assessed using the Student’s t-test and Fisher’s exact test, as appropriate. The normality of the data was assessed via inspection of histograms and the Shapiro–Wilk test.

Differences in SN DTI parameters between the groups were assessed using ANCOVA models, adjusting for age and sex, separately for the SNpr and SNpc. Effect sizes were evaluated using the resulting partial eta squared (partial η2), with >0.01, >0.06, and >0.14 considered small, medium, and large effects, respectively. Receiver operator characteristic (ROC) curves were also calculated separately for FA, MD, AD, and RD in the SNpc and SNpr to evaluate their use as a potential biomarker of PD.

In the PD group, associations between DTI parameters and clinical outcomes (disease duration, UPDRS-III score, and H&Y stage) were assessed using Spearman correlations at baseline.

Correction for multiple comparisons was performed using the Benjamini–Hochberg method [23] to control false discovery rate (FDR). A corrected *p*-value < 0.05 was considered significant while <0.10 was considered as a trend.

## 3. Results

### 3.1. Demographic and Clinical Characteristics

Demographic and clinical characteristics of all study participants are presented in Table 1. No significant differences were detected between PD patients and HCs in terms of age or proportion of males. Of the 14 patients that returned for a follow-up clinical visit, there were no significant differences compared to the 15 that did not with respect to age (*p* = 0.849), sex (*p* = 1.0), UPDRS-III scores (*p* = 0.228), disease duration (0.088), nor H&Y stage (0.156). These patients had a median (interquartile range) change in UPDRS-III score and H&Y stage of 11.5 (5.3–23.0) and 0.5 (0–1.0), respectively.

### 3.2. Differences between Groups

All results are reported in terms of means ± standard deviation. FA is a unitless measure while diffusivity values are reported in 10^–3^ mm^2^/s.

Comparisons between PD patients and HCs are shown in Table 2. Compared to HCs, PD patients presented with significantly increased AD in the SNpc (1.207 ± 0.068 versus 1.156 ± 0.045, pFDR = 0.024), constituting a large effect. This finding was also reflected by a trend for increased MD in the SNpc (0.803 ± 0.054 versus 0.772 ± 0.038, pFDR = 0.08), reflecting a medium effect. No differences emerged in terms of RD nor FA in the SNpc while none were found at all for any of the four measures in the SNpr.

**Figure 2:**
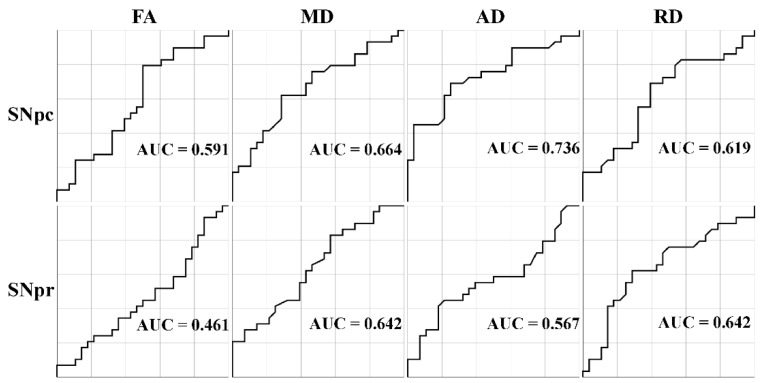
Receiver operating characteristic analysis in the substantia nigra. The top row shows the receiver operating characteristic (ROC) curves for the substantia nigra pars compacta (SNpc) while the bottom row shows ROC curves for the substantia nigra pars reticulata (SNpr). Legend: FA = fractional anisotropy; MD = mean diffusivity; AD = axial diffusivity; RD = radial diffusivity; AUC = area under the curve.

ROC curves are shown in Figure 2. SNpc AD yielded the best performance as a potential biomarker. Full details showing the area under the curve (AUC), standard error (SE), 95% confidence interval (CI), Youden index J, optimal cut-off, sensitivity, and specificity are presented in Table 3. The largest AUC was found for SNpc AD with a value of 0.736, corresponding to a sensitivity of 69.0% and specificity of 75.0%.

### 3.3. Associations with Clinical Outcomes

At baseline, trends were identified for UPDRS III with SNpc MD (rs = 0.449, pFDR = 0.099), AD (rs = 0.388, pFDR = 0.099), and RD (rs = 0.391, pFDR = 0.099) (Figure 2) while no relationship was found with SNpc FA nor for any measure within the SNpr. With respect to H&Y stage and disease duration, no associations were detected for any of the DTI parameters, neither in the SNpc nor in the SNpr. However, a trend was identified between SNpr MD and change in H&Y score over the follow-up in the 14 patients who returned for a clinical visit (rs = 0.563, pFDR = 0.081).

## 4. Discussion

In this study, we utilized a combination of non-linear registration of standard space atlases and conventional DTI-derived quantitative maps to separately assess the SNpc and SNpr in a cohort of PD patients relatively early in their disease. On the one hand, we found that AD within the SNpc yielded the best separation between PD patients and HCs, with a large effect size. Increased AD was also reflected by a trend towards increased MD in the PD patient cohort, whereas no differences were found for RD or FA. The exact interpretation of AD, particularly within the gray matter, is fraught with challenges. However, increased AD is thought to be related to cell degradation and/or loss [12]. Our results shed additional light onto the role that DTI can play in characterizing PD-related SN pathology. Perhaps most importantly, it seems quite certain that having treated the SN as a single structure in previous studies has contributed to inconsistent findings in the literature.

In a recent longitudinal study that implemented a similar approach for measuring magnetic susceptibility changes in the SNpc and SNpr, manual corrections were needed in 17% of the segmentations [24]. In the case of the current study, the border between the SNpc and SNpr was not distinguishable, as expected given the relatively coarse spatial resolution of our diffusion acquisition and inherent limitations of a T2-weighted sequence. Interestingly though, the results from the ROC analysis yielded a very similar AUC with SNpc AD (0.736) to those obtained with R2* measures obtained in the SNpc by warping a neuromelanin atlas in two independent samples (0.730 and 0.751) [25]. While our results appear to be biologically meaningful given the known pathophysiology of PD, it must be stressed that the approach should be independently validated in conjunction with imaging techniques that can specifically visualize the SNpc. Regardless, multi-atlas techniques [26] and/or model-based approaches [27,28] may offer the opportunity to obtain more reliable measurements in a fully automated manner. If DTI-based assessment of the SNpc is to eventually play a role in the diagnosis of PD, such improvements are likely to be essential given that an AUC value of 0.736 is only modest at best and the fact that better sensitivity and specificity would be needed for diagnostic purposes.

While we were able to show robust differences between PD patients and HCs at the group level, our findings with respect to clinical outcomes were somewhat more mixed. We did not find any associations with H&Y stage nor with disease duration in the cohort of patients at baseline. Given that patients using L-Dopa or dopamine agonists were on stable therapy for at least the past three months, one could have reasonably suspected that we would not find associations with the UPDRS-III score due to a potential motor symptom masking effect of treatment. It is not unreasonable to hypothesize that a relationship would have been found if patients had been evaluated in the “off” state. A recent longitudinal study that utilized R2* and quantitative susceptibility mapping, both measures of iron content, in the SN found that their changes over 19 months of follow-up were related to clinical outcomes [24]. This finding suggests that, despite the presence of extensive neurodegeneration within the SNpc already at the time of diagnosis, MRI-derived measures can still inform on ongoing tissue destruction and its relation to clinical progression. Further support for this argument is provided by studies that have shown annual increases of magnetic susceptibility between 3.5% [5] and 5% [24] in the area corresponding to the SNpc. Interestingly though, changes in bi-tensor DTI-derived free-water of the ventral posterior SN, which contains the SNpc, show even larger percent increases, although it tends to deaccelerate over time (12.3% after 1 year versus 6.9% after 4 years) [29]. As of now, studies directly comparing diffusion and iron-sensitive imaging measures in the SN of PD patients are lacking. However, it is reasonable to suspect that combining the two imaging modalities may be even more informative than either of them on their own. Finally, although only about half the PD patient sample returned for a clinical visit, we nevertheless found a moderate association between SNpr MD and change in H&Y score over an average follow-up time of nearly three years. As of now, it is not entirely clear why this finding was limited to the SNpr, whereas more robust findings were found with the SNpc at baseline. Future studies are warranted to confirm or refute our findings. If our findings were to hold up in larger samples, then assessment of the SNpr could potentially be useful for identifying PD patients at risk of clinical progression with appropriate rehabilitative interventions thus being warranted to reduce disease burden.

Our study is not without its limitations. First, our study was conducted on a 1.5T magnet, which may have limited the overall reliability of our results; use of a higher field strength may result in more accurate warping. Moreover, we did not validate our findings using manually drawn ROIs on the *b* = 0 images [9]. It is well-recognized that distinguishing between the SNpc and SNpr is considerably challenging, even with acquisitions using higher spatial resolutions, on most types of sequences. Future studies should confirm our findings using neuromelanin-sensitive imaging scans, which allow for a clearer distinction between the two portions of the SN. In addition, we did not investigate whether free-water corrected DTI metrics, or free-water itself [8] performed better in terms of group separation of PD patients and HCs compared to standard FA, MD, AD, and RD measures. We did not separately evaluate the anterior and posterior portions of the SN structures. Histological studies have demonstrated a gradient whereby damage appears to begin earlier in the posterior region [2]; findings which have been shown also in vivo with both diffusion [29] and magnetic susceptibility [5] imaging modalities. Finally, the cross-sectional nature of the imaging analysis in our study prevented us from establishing the rate of change in AD or from comparing whether there were differences in it compared to healthy controls. Data from the Parkinson’s Progression Markers Initiative (PPMI) cohort, however, suggest that diffusion-related changes deaccelerate over time, at least in terms of increases in free-water accumulation [29]. Future studies should investigate whether an increase in AD precedes subsequent increases in free-water as well as include age and sex-matched healthy controls for longitudinal comparisons.

## 5. Conclusions

Our results demonstrate that the SNpc is characterized by increased AD, potentially reflecting cell damage/loss, and could represent an easily acquired biomarker of PD. The findings from our study suggest that assessment of the SNpc is feasible even without the strict requirement of tailored sequences for its direct visualization. If confirmed, the proposed approach opens the door to the retrospective analysis of historical diffusion datasets to gain further insight into the temporal dynamics of SNpc damage in PD. Finally, additional studies involving both diffusion imaging and iron-sensitive imaging, such as QSM and/or R2*, could be useful to better characterize the extent of damage to the SN. As of now, only a limited number of studies have performed such an analysis. The complementary nature of these imaging modalities though suggests that a combined analysis may potentially be more informative than either measure on its own. Future studies from our group will address this question.

## Figures and Tables

**Table 1:** Demographic and clinical characteristics of study participants.

	PD (*n* = 29)	HC (*n* = 28)	*p*
Age in years, mean (SD)	66.1 (8.1)	65.4 (8.4)	0.742 ^^^
Males, *n* (%)	23 (79.3)	19 (67.9)	0.379 ^%^
Disease duration in years, median (IQR)	2.8 (2.1–5.3)	-	-
UPDRS-III, median (IQR)	21 (8–34)	-	-
H&Y stage, median (IQR)	1.5 (1.25–2)	-	-
LEDD, mean (IQR)	230 (125–325)	-	-

Legend: PD = Parkinson’s disease; HC = healthy controls; SD = standard deviation; IQR = interquartile range; UPDRS-III = Unified Parkinson’s Disease Rating Scale—Part III; H&Y = Hoehn & Yahr; LEDD = levodopa equivalent daily dose; ^ Student’s *t*-test; % Fisher’s exact test.

**Table 2:** Differences in diffusivity measures between PD patients and HCs.

	PD (*n* = 29)	HC (*n* = 28)	Partial η^2^	FDR-Corrected *p*
	Subtantia nigra pars compacta			
FA	0.442 (0.040)	0.432 (0.037)	0.014	0.454
MD	0.803 (0.054)	0.772 (0.038)	0.098	*0.08*
AD	1.207 (0.068)	1.156 (0.045)	0.152	**0.024**
RD	0.601 (0.053)	0.579 (0.045)	0.042	0.454
	Subtantia nigra pars reticulata			
FA	0.565 (0.051)	0.569 (0.046)	0.006	0.582
MD	0.766 (0.033)	0.748 (0.032)	0.070	0.136
AD	1.307 (0.062)	1.290 (0.049)	0.014	0.454
RD	0.495 (0.049)	0.477 (.046)	0.043	0.256

Legend: PD = Parkinson’s disease; HC = healthy controls; FDR = false discovery rate; FA = fractional anisotropy; MD = mean diffusivity; AD = axial diffusivity; RD = radial diffusivity. Cells are displayed as mean (standard deviation). Differences between the group were calculated using an ANCOVA model, correcting for age and sex, and the effect size is presented in terms of partial eta squared. *P*-values corrected for the false discovery rate are shown. Corrected *p*-values < 0.05 are shown in bold; corrected *p*-values < 0.10 associated with original *p*-values < 0.05 (trends) are shown in italics. FA is a unitless measure while diffusivity values are reported in 10^–3^ mm^2^/s.

**Table 3:** Receiver operating characteristic curve analysis.

	AUC	SE	95% CI	pFDR-Value	Youden Index J	Optimal Cut-Off	Sensitivity	Specificity
SNpc								
FA	0.591	0.077	0.428 – 0.730	0.307	0.293	>0.426	79.3%	50.0%
MD	0.664	0.074	0.498 – 0.787	0.102	0.335	>0.782	62.1%	71.4%
AD	0.736	0.067	0.608 – 0.870	0.002	0.440	>1.182	69.0%	75.0%
RD	0.619	0.076	0.510 – 0.808	0.082	0.297	>0.580	69.0%	60.7%
SNpr								
FA	0.461	0.076	0.389 – 0.765	0.172	0.164	≤0.555	48.3%	67.9%
MD	0.642	0.072	0.517 – 0.799	0.082	0.256	>0.741	82.8%	42.9%
AD	0.567	0.076	0.450 – 0.747	0.231	0.235	>1.325	44.8%	78.6%
RD	0.642	0.074	0.519 – 0.808	0.082	0.335	>0.495	62.1%	71.4%

Legend: SNpc = substantia nigra pars compacta; SNpr = substantia nigra pars reticulata; FA = fractional anisotropy; MD = mean diffusivity; AD = axial diffusivity; RD = radial diffusivity; AUC = area under the curve; SE = standard error; FDR = false discovery rate.

## Data Availability

The data that support the findings of this study are available on a reasonable request to the corresponding author (L.P.).
